# Enzymatic Digestion and Selective Quantification of Underivatised Delta-9-Tetrahydrocannabinol and Cocaine in Human Hair Using Gas Chromatography-Mass Spectrometry

**DOI:** 10.1155/2012/907893

**Published:** 2012-04-03

**Authors:** Salah Eddine Breidi, James Barker, Andrea Petróczi, Declan P. Naughton

**Affiliations:** ^1^School of Pharmacy and Chemistry, Kingston University, Penrhyn Road, London KT1 2EE, UK; ^2^School of Life Sciences, Kingston University, Penrhyn Road, London KT1 2EE, UK

## Abstract

Gas chromatography-mass spectrometric (GC-MS) methods for drug analysis routinely employ derivatising reagents. The aim of this paper was to develop a method for the analysis of two recreational drugs, delta-9-tetrahydrocannabinol (Δ^9^-THC) and cocaine in hair samples using GC-MS, without prior derivatisation, thus allowing the sample to be reanalysed in its original form. An enzymatic digestion technique was also developed. Ten hair samples, that were known positive for either Δ^9^-THC and/or cocaine, were enzymatically digested, extracted, and then analysed by GC-MS. All samples measured contained Δ^9^-THC and one sample contained cocaine. The limits of detection (LOD) and quantification (LOQ) were 0.02 ng/mg and 0.05 ng/mg, respectively, for cocaine and 0.015 ng/mg and 0.02 ng/mg, respectively, for Δ^9^-THC. The wide detection window, ease of direct analysis by GC-MS, lower detection limits of underivatised samples, and the stability of drugs using this technique may offer an improved method of analysis.

## 1. Introduction

The United Nation's Office on Drugs and Crimes (UNODC) and the World Health Organisation (WHO) recently estimated that 149–272 million people used psychoactive substances at least once in the past 12 months [1, 2]. The most commonly used substance was cannabis (129–190 million people), followed by amphetamine type stimulants, opioids, and cocaine [1]. Cannabis and cocaine analyses by GC-MS are two of the most frequently used drug assays [3, 4]. Research in analytical sciences has shown a sustained effort to develop methods with improved sensitivity and to facilitate fast, reliable, and cost-effective methods to identify users. The specific reasons behind the need for detection range from current risk to self and others, to future noncompliance [5]. These individually lead to different detection windows. For example, authorities often require evidence of abstinence from drugs before regranting driving licence [6, 7], allowing child custody [8], returning to workplace [9, 10], or licensing to practice [11]. In these cases, the detection window stretches beyond the most recent consumption. The drug detection window is one of the main analytical challenges, since most drugs can only stay in our body system for shorts periods for example, the plasma elimination half-life of tetrahydrocannabinol (THC) and THCCOOH is ca. 4.1 days and 5.2 ± 0.8 days, respectively, for frequent users [12]. Other considerations include the accuracy, reproducibility, sample quantity required, and limits of detection/quantification for a developed method.

Drug analysis in hair has become a very common part of forensic and clinical toxicology (doping, work-place drug testing, rehabilitation programs, and treatment centres) [3, 13, 14]. Hair testing for drugs of abuse is a developing technique that offers the possibility of a longer detection window than is commonly obtained from urine or blood analysis [15, 16] and thus distinguishes between long-term use and short-term single exposure [17]. Hair testing improves drug analysis by being noninvasive; samples are easy to store at room temperature and there is a negligible risk of infection. Hair grows at ca. 1-2 cm each month [3]. Drugs incorporate into hair through several mechanisms, either endogenously, by ingestion (via the blood during hair formation or through sweat and sebum), or exogenously, through external contamination (deposition of drugs on the surface of the hair and passive inhalation) [18]. Due to the extremely low incorporation rate of the psychoactive ingredient of cannabis, (Δ^9^-THC), cocaine, and other drugs in hair (especially in blonde, brown, and thin hair [19]), the development of sensitive techniques is essential for quantitative analysis [14]. The concentration of the drug in hair may reflect the amount of drug used; that is, if the rate of consumption is high or low. In addition, when a sample shows the absence of drug, it does not always mean that the sample is free from the drug, only that the concentration may be too low to be detected. The interpretation of hair analysis involves finding the correct concentrations to distinguish between the common or occasional consumer. Most methods include decontamination, digestion, drug extraction, reconstitution, and derivatisation for sample preparation preceding hair analysis by GC-MS [16].

 In this paper, we report lower limits of detection for Δ^9^-THC and cocaine for the first time *without *derivatisation. This is an improvement on previously reported methods that achieved a lower limit of detection of 2.5 ng/mg for Δ^9^-THC but *with* derivatisation [20]. Further method developments in hair digestion and extraction have also been made, thus permitting direct measurement of the drug without issues such as contamination from reagents, formation of byproducts, and reduction in recovery, chromatographic resolution, and ionisation efficiency that may arise from derivatisation.

## 2. Materials and Methods

### 2.1. Chemicals and Reagents

Delta-9-tetrahydrocannabinol (Δ^9^-THC) and cocaine (1 mg/mL) and their deuterated analogues Δ^9^-THC-D3 and cocaine-D3 (100 *μ*g/mL) were obtained from LGC standards (Teddington, UK). Proteinase K enzyme and HPLC grade pentane were obtained from Sigma Aldrich (Dorset, UK), DTT Cleland's reagent, and TRIS HCl buffer were purchased from VWR, (Leicestershire, UK). Hexane, dichloromethane, and all the other organic solvents were HPLC grade from Fisher Ltd. (Leicestershire, UK).

### 2.2. Sample Preparation

Hair samples (50 mg) were collected from the posterior vertex [21, 22] (posterior cortex hair rarely varies during growth and the hair number in this area is more constant and less affected by age and sex differences [23]) and decontaminated by washing with 3 mL dichloromethane. They were vortex mixed for 2 min (this step repeated 3 times). This cleans the hair from contaminants such as colouring, sebum, shampoo, and so forth, which may interfere with analysis. After decontamination, the hair samples were pulverised to ca. 0.5 mm long segments by hand scissors. The samples were screened for the presence of Δ^9^-THC or cocaine [21, 22].

For enzymatic digestion, the pulverized hair was added to Proteinase K enzyme in a ratio of 1 mg hair: 1 mg enzyme; followed by 100 mg of DTT Cleland's reagent and 1 mL of TRIS HCl buffer. The digestion was undertaken for 50 min at 37.5°C, with continuous mixing. Hair was digested with enzyme in the presence of Δ^9^-THC-D3 and cocaine-D3; a spiked concentration of 1 ng/mg was used as internal standard. Drug-free hair was used as a control. A comparative extraction was conducted using NaOH digestion of spiked hair samples using 1 mL of 1 M NaOH at 95°C for 10 mins followed by neutralisation with 1 mL of 1 M HCl and 3 mL of phosphate buffer (pH = 7, 0.2 M).

The analytes were extracted by liquid-liquid extraction (LLE) using 6 mL of HPLC grade pentane. After vortex mixing and centrifugation (10 min at 2383 × g), the supernatant organic layer was transferred into a fresh glass tube using a Pasteur pipette and the hair residue pellet was discarded. The organic layer was mixed with a 25 *μ*L aliquot of 2 M HCl which had been diluted to 1% in phosphate buffer (pH = 7, 0.2 M) to prevent drug loss during evaporation. The organic layer was evaporated under a gentle stream of nitrogen gas at 50°C using a hotplate concentrator Techno DB-3 (Cambridge, UK). Spiked samples of 0.02–1.50 ng/mg were prepared for the Δ^9^-THC and cocaine calibration plots. The extracted residue was reconstituted with 60 *μ*L hexane, transferred to autosampler vials, and 3 *μ*L was injected into the GC-MS system.

### 2.3. Instrumentation

The extracts were analysed using an AGILENT Technology 7890A gas chromatograph, in combination with an AGILENT 5975 XL EI/CI MSD Triple Axis Detector mass spectrometer connected to a 7683B autosampler (Agilent Ltd., CA, USA) operating in electron impact ionisation (EI) mode using Helium carrier gas with a flow rate of 1.3 mL/min. The analytical column for GC was a BP-X5 SGE Forte Capillary column (Victoria, Australia) (30 m length × 0.25 **μ**m film thickness × 0.25 mm internal diameter) (5% phenyl polysilphenylene-siloxane). Pulsed, splitless injection was performed for a purge time of 1 min, a purge flow rate of 53 mL/min, and an initial pulse pressure of 20 PSI reducing to 15 PSI. This enhanced the peak shape and sensitivity. The GC oven temperature for Δ^9^-THC was programmed to start at 50°C, held for 1 min, then increased to 200°C at 40°C/min, held for 2 min, and increased to 280°C at 80°C/min, held for 3 min, to a final step of 310°C at 80°C/min, held for 4 min.

 For cocaine analysis, the initial temperature was 50°C, held for 1 min, then increased to 200°C at 100°C/min, held for 2 min, then to 280°C at 80°C/min held for 3 min, to a final step of 310°C at 80°C/min, held for 4 min. The injection port temperature was set at 260°C and the transfer line to 280°C. The quadrupole temperature was 150°C; electron multiplier voltage (EMV) 2,200 V. The analysis was performed in selected ion monitoring mode (SIM). The solvent delay time was 7 min; elution window 7–13 min. The precursor and product ions of Δ^9^-THC, Δ^9^-THC-D3 internal standard (IS)_, _cocaine, and cocaine-D3 (IS) were, Δ^9^-THC, *m*/*z* 314, 299; Δ^9^-THC-D3, *m*/*z* 317, 302 cocaine, *m*/*z* 303, 182; cocaine-D3, *m*/*z *306, 185 (precursor ions, product ions). The retention times were 7.9 min and 12.9 min, respectively, for cocaine and Δ^9^-THC. Between samples, at least one drug free sample was analysed to monitor cross-contamination.

### 2.4. Validation

Blank hair (50 mg) was spiked with solutions of the analytes in methanol resulting in calibrator concentrations of 0.02, 0.05, 0.1, 0.5, 1, and 1.5 ng/mg Δ^9^-THC and cocaine and standard curves were linear with *R* ^2^ values of 0.995 and 0.997, respectively. Calibration plots were generated separately for Δ^9^-THC and cocaine. The lower limit of detection (LLOD), lower limit of quantification (LLOQ), intraday precision, interday precision, accuracy, and recovery for each analyte were also calculated. Extraction recovery was determined by comparing the area ratio of Δ^9^-THC and cocaine extracted from 0.5 ng/mg spiked blank hair samples with the area ratio of standard neat solutions prepared in hexane of the same concentration. Cross-contamination was tested by running different blank hair samples. No peaks were noted in the region of both drug's elution times.

## 3. Results and Discussion

### 3.1. Chromatographic Method

**Figure 1:**
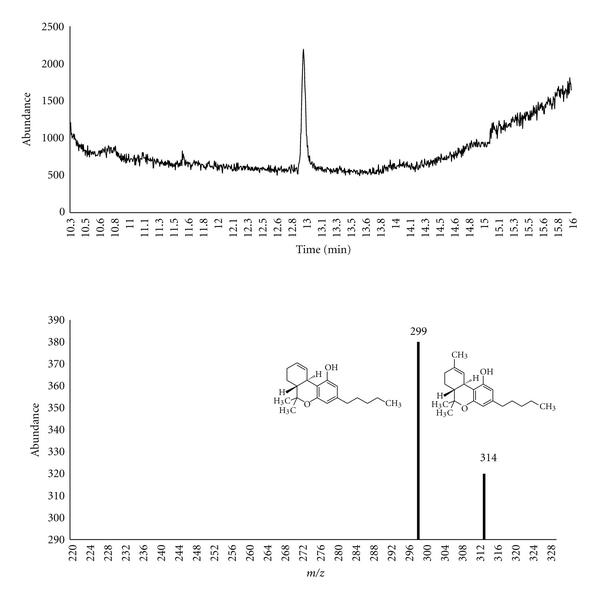
GC-MS chromatogram and fragmentation pathway of Δ^9^-THC.

**Figure 2:**
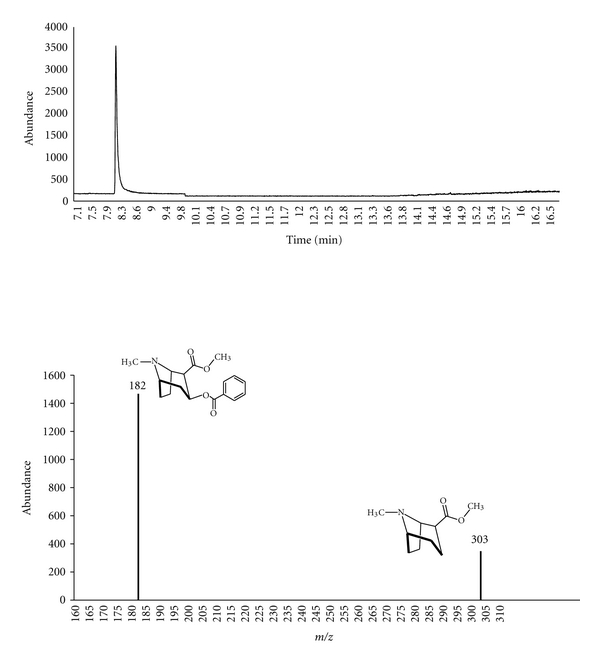
GC-MS chromatogram and fragmentation pathway of cocaine.

Linear calibration graphs (Δ^9^-THC *R* ^2^ = 0.995; cocaine 0.997) were established at concentrations ranging from 0.02–1.00 ng/mg for Δ^9^-THC and 0.05–1.00 ng/mg for cocaine. The calibration curves were prepared by spiking known concentrations of Δ^9^-THC or cocaine to blank hair samples at 0.02, 0.05, 0.10, 0.50, 1.00, and 1.50 ng/mg, with a constant amount of Δ^9^-THC-D3 and cocaine-D3 (1 ng/mg). The calculated peak area from GC chromatograms for spiked concentrations was divided by the peak of the internal standard to determine the abundance ratio. The GC chromatograms (Figures 1 and 2) show peaks representing Δ^9^-THC and cocaine eluting at *R*_*t*_ = 12.9 ± 0.2 and 7.9 ± 0.2 mins, respectively.

### 3.2. Optimisation of the Procedure

**Figure 3:**
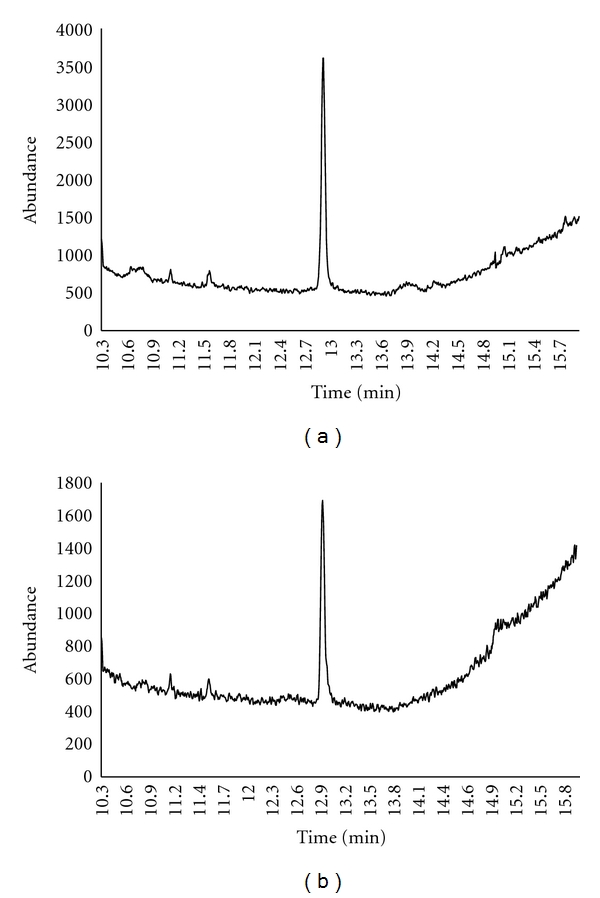
Chromatogram obtained after spiking hair with 5 ng Δ^9^-THC and digesting it with (a) Proteinase K and (b) NaOH.

Results shown in Figure 3 were obtained with the enzymatic and alkaline (NaOH) digestions. For these tests, we intentionally used a blank hair sample to reproduce true working conditions. Δ^9^-THC (5 ng/mL) was spiked into two blank hair samples and digested with Proteinase K at 37°C or by NaOH at a temperature not greater than 95°C. This process was repeated 6 times for each digestion method. Use of Proteinase K resulted in an average concentration of 4.85 ng/mL ± 0.23 with 95% recovery, whilst the samples digested by NaOH resulted in a mean of 3.15 ng/mL ± 0.1 and 62% recovery.

One of the explanations for the reduced extraction recovery when using NaOH is the drug degradation caused by the strong basic NaOH conditions and high temperature. This enzymatic hydrolysis method is an improvement over other approaches, which could easily cause drug degradation in the presence of sodium hydroxide (NaOH), hydrochloric acid (HCl) and high temperatures, and thus improve the stability of the method and therefore accuracy.

### 3.3. Method Validation

Validation results are shown in Table 1. In the spiked hair samples, detection was feasible for Δ^9^-THC concentrations as low as 0.015 ng/mg and for cocaine at 0.02 ng/mg and quantification was possible at the concentrations of 0.02 ng/mg and 0.05 ng/mg for Δ^9^-THC and cocaine respectively using a signal to noise ratio >3.

### 3.4. Real Sample Analysis

All qualitatively identified positives (samples prescreened by ELISA; 9 positive for only Δ^9^-THC and 1 positive for both Δ^9^-THC and cocaine) were confirmed and quantified by GC-MS. Ten hair samples were positive for Δ^9^-THC and one also for cocaine (Table 2). Using the abundance ratio, the actual drug concentrations (ng/mg) in the unknown hair samples ranged between 0.05–0.35 ng/mg hair for Δ^9^-THC. The only positive sample for cocaine was measured to be 0.1 ng/mg.

The number of publications describing analytical procedures relating to drug incorporation into hair, decontamination and analysis has increased in recent years [2, 23–26], but in this paper an improved digestion and GC-MS method has been proposed which enhances drug analysis capabilities. The sensitivity achieved for Δ^9^-THC (LOD 0.01 ng/mg ± 0.01 and LOQ 0.02 ng/mg ± 0.01) and for cocaine (LOD 0.02 ng/mg ± 0.015 & LOQ 0.05 ng/mg ± 0.01) is better than previous reports which have been obtained from derivatised samples (LOD 0.025–2.5 ng/mg, LOQ 0.05–7.5 ng/mg for Δ^9^-THC and LOD 0.03–0.5 ng/mg, LOQ 0.05–1 ng/mg for cocaine) which is higher than what we achieved [20, 27–30]. Negative results could also mean that the concentration of drug in hair is below the detection limit of the method. An additional advantage of the increased sensitivity is that the analysis required a reduced amount of hair, thus making the method more feasible for drug testing [31].

Although derivatisation is an important factor to improve sensitivity, it can be problematic in complex matrices. Derivatisation is sometimes time consuming, can add possible contamination to the sample mixture, and could result in a decrease in the sensitivity of the method. Also, derivatisation can create new interfering degradation product ions of the drug itself [32]. The performance in terms of reliability, feasibility, and length of analysis can be improved by using enzymes. In this study, GC-MS has been performed on underivatised, informed positive hair samples for cocaine and Δ^9^-THC. All the 10 samples in Table 2 were positively confirmed (by ELISA) for Δ^9^-THC (10 samples) and 1 sample was found to be also positive for cocaine.

## 4. Conclusions

Hair analysis has proved to be a useful method of public health research in the case of Δ^9^-THC and cocaine screening. The method that has been developed is capable of detecting exceptionally low levels of Δ^9^-THC and cocaine in human hair when only ca. 50 mg hair was processed. Enzymatic digestion and the given chromatographic and mass spectrometric conditions were essential for reproducible and accurate analysis of these psychotropic drugs in hair without any interference. An additional advantage of this method is that unlike previously published work, it does not require derivatisation [17, 20, 23, 24]. Thus, it is a convenient and potentially less problematic method which can be employed for routine drugs testing. This method can complement conventional blood and urine analysis with the advantages of noninvasiveness of sample collection, negligible risk of infection (blood analysis), facile sample storage (small sample size, limited biohazard and adulteration/contamination risks), and negligible sample degradation. Also, hair analysis could help prevent false negative ELISA results that can be encountered from the higher limit of detection or even previously developed GC-MS methods by detecting ultra-low concentrations. The limit of detection for ELISA screening was found to be 0.5 ng/mg for the cocaine kit and 0.3 ng/mg for Δ^9^-THC. These were used as cut-off levels for screening of the hair samples.

## Figures and Tables

**Table 1:** Summary of assay validation results.

Compounds	Recovery (%) at 0.5 ng/mg (*N* = 6)	Concentration (ng/mg)	Precision RSD (%)		Accuracy (%)
			Intraday *n* = 6 per each	Interday *n* = 18 per each	
Δ^9^-THC		0.02	15.4	18.0	114
		0.10	15.6	7.0	110
	102.2	0.50	8.0	5.0	95

Cocaine		0.05	12.1	10.6	98
		0.10	10.1	12.1	101
	96.5	0.50	5.1	2.8	110

**Table 2:** Hair analysis results of samples using GC-MS.

Hair samples	Age	Gender	Δ^9^-THC ng/mg	Cocaine ng/mg	SD	SEM
					*n* = 3	
H1	22	F	0.08	ND	±0.006	0.003
H2	22	F	0.05	ND	±0.001	0.001
H3	18	F	0.35	ND	±0.006	0.003
H4	21	M	0.20	ND	±0.000	0.000
H5	24	M	0.18	ND	±0.017	0.010
H6	22	M	0.09	ND	±0.006	0.003
H7	20	M	0.08	ND	±0.010	0.006
H8	27	M	0.14	ND	±0.012	0.007
H9	18	M	0.13	ND	±0.006	0.003
H10	23	M	0.15	0.1	±0.00/±0.015	0.000/0.009

ND: not detected, SD: standard deviation, SEM: standard error of mean.
